# Optimization strategy of community planning for environmental health and public health in smart city under multi-objectives

**DOI:** 10.3389/fpubh.2024.1347122

**Published:** 2024-02-14

**Authors:** Fujiang Chen, Wuyue She, Feng Zeng, Meiben Gao, Chao Wen, Guoxin Liu, Lixun Wu, Yang Wu, Chi Zhang

**Affiliations:** ^1^School of Emergency Management, Xihua University, Chengdu, China; ^2^National Dam Safety Research Center, Wuhan, China; ^3^School of Public Administration, Southwestern University of Finance and Economics, Chengdu, China; ^4^The Research Center for Social Development and Social Risk Control of the Key Research Base of Philosophy and Social Sciences in Sichuan Province, Chengdu, China; ^5^State Key Laboratory of Geomechanics and Geotechnical Engineering, Institute of Rock and Soil Mechanics, Chinese Academy of Sciences, Wuhan, China

**Keywords:** environmental hygiene, public health, land use, service facilities, community planning

## Abstract

As population density increases, environmental hygiene and public health become increasingly severe. As the space where residents stay for the longest time and have the most profound impact on their physical and mental health, the quality of the environment in urban communities largely determines the degree to which residents engage in physical activity, bear the risk of pollution exposure, and obtain healthy food. Therefore, in order to ensure the physical and mental health of residents, this study proposes community planning guided by environmental hygiene and public health, and establishes an environmental health assessment system for this purpose. This system evaluates the community environment from four aspects: land use, service facilities, site convenience, and environmental quality. Established the diversity, density, road network connectivity and facilities accessibility nine criteria, as well as the land function of mix, plot ratio, food environment, network ring α and connected β index, pavement risk level, green configuration and neighborhood material environment disorder degree of 27 indicators of community built environmental evaluation index system. The data is collected through field survey, questionnaire distribution, resident interview and data mapping, and the established evaluation index system is used to evaluate the construction environment of the community. The experimental research data included population data, CAD plan, land use data, street data, POI point data, building data and bus station data, etc. 273 questionnaires were distributed, 264 were recovered, 8 invalid questionnaires were removed, and 256 valid questionnaires were obtained. These experiments confirm that land use, service facilities, site convenience, and environmental quality have a significant impact on the built environment of communities, with impact weights of 0.513, 0.227, 0.135, and 0.125, respectively. The above weights are calculated based on the index judgment matrix and the eigenvectors. The scores of land use, service facilities, site convenience, and environmental quality for the study subjects were 3.44, 1.46, 0.94, and 0.51, respectively, among them, the land use score is less than 3.85, the 1 service facility score is less than 1.71, the site convenience score is less than 1.01, and the environmental quality score is less than 0.94; indicating that the community has serious problems such as single land use types, pollution exposure, and difficulty in obtaining healthy food. Therefore, community planning and transformation based on land use, service facilities, venue convenience, and environmental quality can effectively improve the physical and mental health of residents. In the specific community transformation plan, artificial intelligence and data-driven methods can be used to optimize the land use plan, service facility configuration, site convenience transformation and environmental quality improvement, so as to formulate the optimal community transformation plan and improve the comfort and happiness of community residents. In the future, on the basis of the existing research, the selection of community types will be further enriched and the research cases will be expanded. And through the in-depth practical study of the case, the constructed evaluation index system is optimized and improved to make it more scientific. At the same time, as urban renewal and design have entered the era of stock planning, based on the more perfect evaluation index system, more specific and detailed system discussion of the built communities with public health problems, in order to provide more detailed services for the construction of a better and healthy living environment in the future.

## 1 Introduction

Since the First Industrial Revolution, the scale and function of cities have developed rapidly, providing convenient lifestyles for residents. With the influx of a large population into cities, the transportation and energy systems of the city are overwhelmed and almost collapsed. Smart cities can make key infrastructure components and services composed of cities such as urban management, education, healthcare, real estate, transportation, public utilities, and public safety more interconnected, efficient, and intelligent through the application of technology (1, 2). These technologies include intelligent computing such as the Internet of Things (IoT), cloud computing, big data, and spatial geographic information integration. Smart cities have greatly alleviated the operational pressure of the city and provided residents with a more convenient and efficient way of life. For example, through the construction of the basic network of things covering the whole city, intelligent transportation, electronic medical records, telemedicine, intelligent home, e-commerce can be realized, so as to make the urban services more intelligent and humanized. Although smart cities provide a great variable for people’s lives, excessive convenience leads to a sharp decline in people’s activity, while a large number of buildings squeeze people’s activity space, leading to a rapid increase in obesity, cardiovascular disease and mental disease, posing a serious threat to public health. In addition, research has found that the incidence rate of cancer is closely related to the process of urbanization. As a long-term living environment for residents, community environment has a significant impact on their health. Gunn (3), Tang et al. (4), and Ram et al. (5) have shown that many chronic noncommunicable diseases are closely related to urban life, and a high quality of living environment can effectively improve residents’ ability to prevent chronic noncommunicable diseases and their mental health. Currently, research on community environment and health is mostly focused on individual health such as “environmental satisfaction,” “happiness,” “residents’ physical and mental health,” and “sense of security,” with less research on public health. In addition, due to the impact of industrialization, the current environmental pollution in cities is prominent, and the number of urban communities is large, many communities are close to production areas, and the decline of the built environment has a negative impact on the public health of residents to varying degrees. And these problems have not been systematically combed, and need to be weighed by a scientific and perfect evaluation system. Therefore, in order to explore the relationship between community environment and public health, construct the evaluation system of scientific community environment, and guide the design practice of community optimization strategies, the study establishes an environmental health evaluation system through Analytic Hierarchy Process (AHP) and 5Ds theory. This study explores the relationship between community environment and health from a novel perspective, providing strong reference for the creation of a healthy urban environment. The research focuses on the connotation cognition and influence elements of the community building environment that promote public health, and constructs a systematic and scientific community building environmental evaluation system, which can further supplement the research direction and content of community building environmental evaluation in theory. At the same time, it provides reliable countermeasures and innovative ideas for the optimization and improvement of the community built environment and the construction of urban healthy community and healthy city, and provides reference cases for the environmental optimization of public health oriented community under the background of urban renewal.

The study consists of four sections, starting with a description of relevant research on community environment and public health. Secondly, the environmental health assessment system is constructed. Section 3 conducts empirical analysis on the environmental health assessment system and proposes optimization methods. Finally, a summary of the entire research is provided.

## 2 Related works

Due to changes in lifestyle and deterioration of the natural environment, people are facing increasingly severe public safety crises. To explore the impact of urban green space on residents’ physical and mental health, Guan et al. conducted analysis using methods such as ArcGIS analysis and field surveys, and established a regression model. These experiments confirmed that small public green spaces near densely populated areas had a greater impact on promoting public health activities than those located on densely mixed land (6). Wang et al. conducted a multi-scale analysis on the impact of air pollution and economic conditions on public health by using multiple ordered Logit model and panel data regression model. These experiments confirmed that economic level was positively correlated with public health, while air pollution was negatively correlated with public health (7). Surzhikov et al. evaluated the impact of air pollution on the health of urban residents through factor analysis, multiple regression analysis, and discriminant analysis. These experiments confirmed that the calculated concentration of atmospheric pollutants exceeded the acceptable cancer risk threshold. Therefore, air pollution would increase the risk of cancer among residents (8). Minovi conducted an analysis of the BioLab chlorine plant fire in Lake Charles, Louisiana to investigate the impact of extreme weather induced chemical releases on public health. He found that extreme weather conditions such as hurricanes had led to more frequent release of chemicals. This increased the exposure of nearby communities to hazardous chemicals, posing a serious threat to the safety and health of residents (9). Rajaee et al. proposed a method to reduce lead absorption by supplying calcium fortified drinking water in response to public safety issues caused by lead exposure. These experiments confirmed a significant negative correlation between biomarker lead levels and calcium intake or serum calcium, with a concentration of 60 milligrams of calcium per liter providing 22.0 and 16.3% of daily calcium RDA for men and women, respectively (10).

The expansion of cities has gradually changed people’s lifestyles, and the changes in urban environment have a great impact on residents’ health, safety, and other aspects. Ram et al. conducted a study on the impact of changes in the architectural environment on mental health and subjective well-being. In this study, a building environment that was closer to the park and had better public transportation was more conducive to improving residents’ neighborhood perception (5). Barnett et al. conducted a study on the relationship between adolescent obesity and community environment through multivariate linear regression analysis. These experiments confirmed that residents of moderately walkable/low safety communities had higher FMI and waist circumference. Therefore, neighborhoods with lower traffic safety seemed to be the places where children were most likely to become obese (11). Basu et al. conducted a survey on the impact of built environment on pedestrian perception of walking environment. These experiments confirmed that women generally believed that leisure areas and open spaces had a higher sense of security compared to residential areas, while young people believed that areas with commercial and mixed land use had a higher sense of security (12). Kanwal and Khan conducted research on the impact of building environment on urban climate. They found that as population increased, urban green space continued to decrease, high-rise buildings continued to increase, and urban temperatures also continued to rise. The changes in urban temperature patterns might be due to high energy consumption caused by population growth and lifestyle changes, which would be the main source of carbon dioxide emissions (13). Yin et al. evaluated the impact of building environment on transportation modes using the GSEM model. These experiments confirmed that compared to community building environments, urban building environments had a stronger correlation with active commuting time, BMI, and life satisfaction, but a weaker correlation with active commuting patterns (14). Zielinska-Dabkowska et al. have studied the impact of urban luminous pollution on human health. Light at night can strain the visual system, disrupt the physiological rhythm, inhibit the secretion of melatonin, and affect sleep. Increasing work points to the adverse effects of outdoor lighting on human health, including an increased risk of chronic disease (15).

In summary, there have been many achievements in research on community environment and public monitoring. But there is little research on evaluating community environment from the perspective of public health. Therefore, the study constructs a community environmental assessment system guided by public health through AHP, providing reference for community environmental planning in smart cities.

## 3 A community environmental assessment system guided by environmental hygiene and public health

In urban planning, design, and construction, intelligent technologies such as IoT, cloud computing, big data, and spatial geographic information integration can achieve the interconnection and intelligence of various infrastructure, improving the construction foundation for smart cities. But in the construction of smart city, public health is a key factor that cannot be ignored. The historical process of urban planning is inseparable from public health. The origin and purpose of modern urban planning is to improve public health, so as to meet the needs of social reform. However, in the process of smart city construction, the city scale will inevitably expand, and thus occupy the previously unexplored ecosystem, increasing the risk of human exposure to new pathogens; and the rising population density promoted by smart city growth is likely to become the cradle and catalyst for the spread of infectious diseases. Among the influencing factors of urban public health, the community environment is a key factor that cannot be ignored. As the space where people have the longest contact time, the community environment has an important impact on people’s lifestyle, food intake and physical activity, and indirectly affects public health. However, it is currently difficult to establish universal evaluation standards for the impact of community environment on public health. At present, most of the research on the impact of the environment on public health and its evaluation system focuses on the developed urban communities with high urbanization level and fast pace of life, and the evaluation index system is not universal. At the same time, the elements of the existing norms and standards are too broad, including the construction environment, cultural environment, service elements, etc., and do not focus on the construction environment, which is not conducive to the development of the environmental health research of the community construction. Therefore, in order to realize the multi-scale and multi-objective perspective evaluation of environmental health in smart cities, the “5Ds” theory is adjusted and improved by the principle of public health orientation, so as to obtain the completed environmental elements that affect public health, and on this basis, a public health-oriented community environmental evaluation system with public health is established.

### 3.1 Extraction of influencing factors for community environmental construction

The ontological characteristics of a community include issues such as blocks, road networks, and scales. To accurately measure the built environment of a community, research focuses on five aspects: diversity, density, design, public transportation distance, and destination accessibility to screen community environmental elements. Starting from two different scales of micro and macro, feature element indicators are selected in different levels for land use, environmental quality, service facilities, and convenience of places. Table 1 shows the characteristic elements of land use, environmental quality, service facilities, and site convenience.

**Table 1:** Characteristics of the different aspects.

Aspects	Characteristic element	Function
Land use	Land-use function mix degree	To evaluate the rationality and richness of the land use layout
	Pollution exposure	Outdoor activities are free from noise, odor, waste water and air pollution measurement
	Density of population	Number of population per unit of land area
	Building density	Within a certain range, the sum of the base area of the building and the proportion of the occupied area
	Plot ratio	For comprehensive rational elaboration density index
Service facility	Facility location	Evaluate whether the layout of various facilities is reasonable
	The number of facilities	Whether the quantity meets the living needs of the residents
	Facilities use	The use of various facilities by the residents
Place convenience	Connexity	To evaluate the path to the destination
	Accessibility	Evaluate the difficulty to reach the destination
Environmental quality	Security	To evaluate the improvement of criminal safety, pavement installation and accessibility facilities
	Beauty	Evaluation of intelligent environmental order, landscape design, architectural design, comfort and other factors

**Figure 1:**
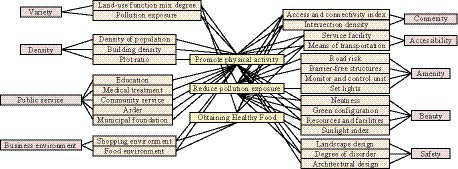
The impact of various environmental elements on public health.

In Table 1, land use includes five elements: mixing degree, population density, building density, plot ratio, and pollution exposure. The mixing degree of land use functions is the foundation for residents to generate physical interaction. Service facilities include three elements: facility location, quantity, and usage, all of which are closely related to the degree of functional mixing of land use (16, 17). Place convenience includes two elements: connectivity and accessibility, with connectivity being the foundation of accessibility. Environmental quality includes two elements: safety and aesthetics. The above evaluation indicators each have their own focus, but they all affect public health by reducing pollution exposure, obtaining healthy food, and promoting physical labor. Figure 1 shows the impact of various environmental factors on public health.

In Figure 1, basic environmental factors will have an impact on promoting physical activity. Transportation facilities, road network connectivity, and connectivity index will have an impact on physical activity, pollution exposure risk, and access to healthy food. The mixing degree of land use and commercial environment will have an impact on both physical activity and access to healthy food. Pollution exposure, intersection density, service facilities, green configuration, cleanliness, resource facilities, and environmental disorder will have an impact on physical activity, pollution, and human exposure risks. For urban construction, its core is land use. The impact of land use on urban public health includes both diversity and density (18–20). Diversity is mainly measured through two indicators: land use mixing degree and pollution exposure degree. Equation 1 is the calculation of land use mixing degree.


(1) $$H\left(X\right)=-\overset{n}{\underset{i=1}{\sum }}P_{i}\log P_{i}$$

In Equation 1, $H\left(X\right)$ represents the mixed entropy of land use. $P_{i}$ represents the probability that the random variable X takes the value of $X_{i}$ . The high degree of land use mixing indicates that the community has multiple types of land use, balanced area distribution, and balanced distribution of various facilities, which can provide richer services for community residents and promote the generation of leisure physical activities. The evaluation of pollution exposure is generated through community residents scoring, with high scores indicating low pollution exposure. Density is evaluated by three indicators: population density, building density, and plot ratio, where plot ratio is the quotient of total building area and total land area. A high density means a high level of improvement in various infrastructure, a smaller demand area for residents’ daily life, work, and leisure, shorter commuting distances, and a higher likelihood of generating transportation related physical activities. Excessive density can also lead to issues such as limited private space, slow air circulation, and accumulation of pollutants, which can have a negative impact on residents’ physical and mental health. Community service facilities are divided into two categories: public service facilities and commercial service facilities, among which public service facilities include education, healthcare, leisure, community services, and municipal infrastructure (21, 22). Commercial service facilities have a significant impact on residents’ physical activity and food access, and Table 2 shows their environmental factors.

**Table 2:** Business environment elements and subsystem connotation.

	Type	System connotation
Business environment	Shopping environment	Shopping malls, daily grocery stores, hardware stores, stationery points, retail and wholesale stores, etc
	Food environment	Stores prone to obesity and health hazards (fast food, candy, dessert, barbecue, tobacco and alcohol retailers)
		Medium is easy to lead to obesity in restaurants (Most other restaurants/ non-fast food)
		Shops that are less likely to cause obesity (supermarkets, fruit shops, vegetable markets, agricultural and sideline products markets, etc.)

In Table 2, in a commercial environment, food has a decisive impact on the types of food consumed by residents. Research has found that residents who are closer to supermarkets have a lower obesity index. This is because the closer you are to the supermarket, the healthier the types of food residents consume. To accurately reflect the richness and quantity of service facilities, the study uses ground average density to measure them. The convenience of a place is measured by its connectivity and accessibility, both of which are closely related to the road. Connectivity refers to the number of roads per unit area connecting the destination, which is measured by two indicators: network connectivity and connectivity, which are calculated using Equation 2.


(2) $$\left\{ \begin{array}{l} \alpha =\frac{L-V+1}{2V-5}\\ \beta =\frac{L}{V}\left(\alpha \in \left[0,1\right]\right) \end{array}\right.$$

In Equation 2, α represents the road network connectivity index. L represents the number of roads connecting the destination. V represents the number of nodes in the road network. β represents the connectivity index of the road network. The closer the road network connectivity index is to 1, the larger the road network loop. A high connectivity index indicates a high level of connectivity between community roads and urban roads. The accessibility of a place refers to the proximity of a residence to services and transportation facilities, which is mainly measured by the number of services and transportation facilities. Environmental quality is mainly measured by safety, aesthetics, and comfort, among which safety is mainly used to measure community security environment and road safety risks. Aesthetics is mainly used to measure landscape design and environmental hygiene, and good aesthetics are beneficial to the physical and mental health of residents. Comfort is used to measure the green configuration, cleanliness, and sunlight indicators of a community (23, 24). Considering that there are not only objective indicators of environmental quality, but also subjectivity in residents’ choices, the Likert scale method was used to evaluate it in this study. By improving the “5Ds” theory, although the key elements of the community building environment can be effectively extracted, how to determine the weight of each element is a major difficulty. If the weight setting is not reasonable, the reliability of the research will be greatly reduced.

### 3.2 Construction of a community environmental assessment system based on environmental hygiene and public health

Scholars have adopted different methods to construct an evaluation system based on environmental hygiene and public health. But because of its inherent sensitivity and uncertainty, the entire evaluation system may collapse due to the instability of a certain subsystem. Take the construction of the environmental evaluation system as an example, if the steady state of the land use evaluation system is unbalanced, it will cause the imbalance of other subsystems, and then lead to the collapse of the whole evaluation system. Therefore, to construct a scientific and reasonable evaluation system, the study constructs a subsystem by merging and calculating various elements, and assigns weights to each criterion layer using AHP to achieve objective evaluation of community environmental health (25, 26). Hierarchical analysis refers to a complex multi-target decision problem as a system, the target into multiple goals or criteria, and decomposed into multiple indicators (or criteria, constraints) of several levels, through qualitative index fuzzy quantitative method to calculate level single sort (weight) and total sort, as a target (indicators), more optimization decision system method. Table 3 shows the evaluation index system of environmental factors.

**Table 3:** Evaluation index system of environmental elements.

Target layer	Standard layer	Index layer	Measure the content	Method	Data sources
Land use	Variety	Land-use function mix degree	Land type	GIS	Field research
		Pollution exposure	Garbage station, sewage treatment station, small three-class industry	Questionnaire survey	Questionnaire survey
	Density	Density of population	Population number, land area	GIS	The seventh census, municipal statistical Yearbook
		Building density	Building outline, land area	GIS	Baidu Map
		Plot ratio	Building outline, building height, land area	GIS	Baidu Map
Service facility	Public service	Education	Location, quantity and use of schools, cultural centers, libraries, etc	GIS and questionnaires	POI, field research
		Medical treatment	Location, quantity, and use of hospitals, pharmacies, and clinics	GIS and questionnaires	POI, field research
		Community service	Location, quantity, and use of community service centers	GIS and questionnaires	POI, field research
		Arder	Location, quantity and use of green space, parks, squares, etc	GIS and questionnaires	POI, field research
		Municipal foundation	Location, quantity and use of water supply / drainage facilities and sanitation facilities	GIS and questionnaires	POI, field research
	Business environment	Shopping environment	All kinds of shopping places	GIS and questionnaires	POI, field research, and Baidu Map
		Food environment	Location, quantity and use of all kinds of restaurants and food sales stores	GIS and questionnaires	POI, field research, and Baidu Map
Place convenience	Connexity	Road network loop access and connectivity index	The actual number of connections and nodes in the road network	GIS	Baidu Map
		Intersection density	Number of intersections and the total land area	GIS	Baidu Map
	Accessibility	Service facility	Access to public services and to the food environment	GIS	Baidu Map
		Means of transportation	Location of bus stations, types of public transportation, parking location and scale	GIS	Baidu Map
Environmental quality	Safety	Road risk	Pavement grade, sidewalk proportion, etc	Questionnaire, GIS	Questionnaire survey, Baidu map
		Barrier-free structures	Type and quantity of barrier-free facilities	Research statistics	Field research
		Monitoring equipment	Number of equipment, total land area	Research statistics	Field research
		Set lights	Number of lighting equipment and total land area	Research statistics	Field research
	Amenity	Neatness	The influence degree and frequency of odor, noise, etc	Questionnaire, interview	Questionnaire survey, field research
		Green configuration	Type and scale of green plants	Questionnaire, interview	Questionnaire survey, field research
		Resources and facilities	The location, quantity and use of public toilets and fitness equipment	Questionnaire, interview	POI, field research
		Sunlight index	Building height and spacing	GIS	Baidu map
	Beauty	Landscape design	Location, quantity and use of sculpture and other landscape designs	Questionnaire, interview	POI, field research
		Degree of neighborhood environment disorder	The extent of random graffiti, littering, etc	Questionnaire, interview	Field research
		Architectural design	Evaluation of architectural color, height, style, etc	Questionnaire	Questionnaire survey, field research

The GIS represents the geographic information system, and the POI indicates the points of interest.

**Figure 2:**
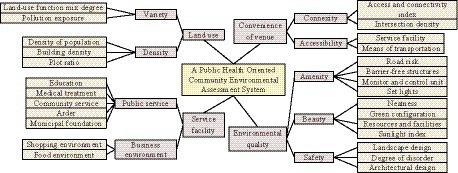
Hierarchy of weight determination.

In Table 3, the environmental quality has the most element indicators among the target layers, as it depends more on the subjective feelings of residents and involves more complex elements compared to other target layers. In the establishment of environmental evaluation index system, the reason why pollution exposure is selected as the index of land use evaluation is because the human body is exposed to the polluted environment for a long time, and harmful substances will enter the body through human respiratory tract, digestive tract or skin contact and harm health. The evaluation of community pollution land can effectively reflect the degree of community pollution and its harm to health. To scientifically and reasonably evaluate the impact of community environment on public health, the study will assign weights to each criterion and indicator layer through AHP (27). Figure 2 shows the hierarchical structure of weight determination.

In Figure 2, the entire evaluation system is divided into objective, criterion, and indicator layers. The target layer includes land use, service facilities, site convenience and environmental quality; the criterion layer includes land use diversity, density, public service, business environment, road network connectivity, facility accessibility, safety, comfort and aesthetics. Based on the importance of indicators at each level, research is conducted to rate them based on expert questionnaire surveys and construct a judgment matrix. 1. 3, 5, 7, and 9 are important, slightly important, more important, strongly important, and extremely important, respectively, while 2, 4, 6, and 8 are the intermediate values of adjacent judgments (28–30). Table 4 shows the judgment matrices for each layer.

**Table 4:** The judgment matrix of each layer.

Target layer				
	Land use	Service facility	Place convenience	Environmental quality
Land use	1	3	3	4
Service facility	1/3	1	2	2
Place convenience	1/3	1/2	1	1
Environmental quality	1/4	1/2	1	1

Standard layer		
Land use	Variety	Density
Variety	1	3
Density	1/3	1

Service facility	Public service	Business environment
Public service	1	2
Business environment	1/2	1

Place convenience	Connectivity	Place accessibility
Connectivity	1	1
Place accessibility	1	1

Environmental quality	Safety	Amenity	Beauty
Safety	1	1/2	3
Amenity	2	1	3
Beauty	1/3	1/3	1

Index layer		
Variety	Land-use function mix degree	Pollution exposure
Land-use function mix degree	1	2
Pollution exposure	1/2	1

Density	Density of population	Plot ratio	Building density
Density of population	1	5	1/3
Plot ratio	1/5	1	1/7
Building density	3	7	1

Public service	Cultural education	Medical treatment	Community service	Arder	Municipal foundation
Cultural education	1	2	2	1/2	2
Medical treatment	1/2	1	3	1/2	1/2
Community service	1/2	1/3	1	1/2	1/2
Arder	2	2	2	1	2
Municipal foundation	1/2	2	2	1/2	1

Business environment	Shopping environment	Food environment
Shopping environment	1	1/2
Food environment	2	1

Connectivity	Road network loop access and connectivity index	Intersection density
Road network loop access and connectivity index	1	2
Intersection density	1/2	1

Place accessibility	Service facility	Means of transportation
Service facility	1	2
Means of transportation	1/2	1

Safety	Road risk	Barrier-free structures	Lighting installation	Monitoring equipment
Road risk	1	5	3	2
Barrier-free structures	1/5	1	2	1/2
Lighting installation	1/3	1/2	1	1/2
Monitoring equipment	1/2	2	2	1

Amenity	Neatness	Sunlight index	Green configuration	Resources and facilities
Neatness	1	1/4	3	1
Sunlight index	4	1	4	2
Green configuration	1/3	1/4	1	1/3
Resources and facilities	1	1/2	3	1

Beauty	Landscape design	Degree of neighborhood environment disorder	Architectural design
Landscape design	1	1/2	3
Degree of neighborhood environment disorder	2	1	3
Architectural design	1/2	1/3	1

In Table 4, the importance relationship between each indicator can be determined based on the above judgment matrix, and the weights and eigenvectors of each indicator can be obtained. Based on the eigenvectors, the maximum feature root can be calculated. Equation 3 is the calculation of the maximum characteristic root.


(3) $$\lambda_{\text{max}}=\overset{n}{\underset{i=1}{\sum }}\frac{{\left(\mathrm{AW}\right)}_{i}}{nW_{i}}$$

In Equation 3, $\lambda_{\text{max}}$ represents the maximum feature root. A represents the judgment matrix. $W_{i}$ represents the feature vector. n represents the dimension of the matrix. According to the maximum feature root, $C_{i}$ used for consistency test in Equation 4 can be obtained.


(4) $$C_{i}=\frac{\left(\lambda_{\text{max}}-n\right)}{n-1}$$

Equation 5 is the consistency indicator used to evaluate the weights.


(5) $$CR=\frac{C_{i}}{R_{i}}$$

In Equation 5, CR represents the consistency indicator. $R_{i}$ represents the average random consistency indicator. The smaller the value of CR indicates that the better the consistency of the judgment matrix. When CR is less than 0.1, the judgment matrix is consistent, otherwise, the judgment matrix of the index needs to be adjusted mechanically. Table 5 shows the weights for each level.

**Table 5:** Weights of each level.

Target layer	Weight	Standard layer	Weight	Index layer	Weight
Land use	0.513	Variety	0.384	Land-use function mix degree	0.256
				Pollution exposure	0.128
		Density	0.128	Density of population	0.036
				Plot ratio	0.010
				Building density	0.082
Service facility	0.227	Public service	0.152	Education	0.036
				Medical treatment	0.024
				Community service	0.016
				Arder	0.048
				Municipal foundation	0.028
		Business environment	0.076	Shopping environment	0.025
				Food environment	0.051
Place convenience	0.135	Connectivity	0.068	Road network connectivity and loop communication index	0.045
				Intersection density	0.023
		Accessibility	0.068	Service facility	0.045
				Means of transportation	0.023
Environmental quality	0.125	Safety	0.042	Road risk level	0.02
				Barrier-free structures	0.007
				Monitoring equipment	0.01
				Lighting equipment	0.005
		Amenity	0.066	Neatness	0.013
				Green configuration	0.006
				Sunlight index	0.032
				Resources and facilities	0.015
		Beauty	0.018	Landscape design	0.006
				Degree of neighborhood environment disorder	0.009
				Architectural design	0.003

The weights of the above indicators at all levels have passed consistency testing.

In Table 5, the weight indices of each level have passed the consistency test, indicating that the weight configuration is reasonable and can be used to evaluate environmental health. In each evaluation index, the weights of land use, service facilities, site convenience and environmental quality are 0.513, 0.227, 0.135, and 0.125, respectively. In land use, the largest weight proportion is the mixed land use function, whose weight is 0.256. Among the service facilities, the food environment weight accounted for the largest proportion of 0.051. Among the site convenience, the weight of road network connection and loop communication index and service equipment is the largest, both are 0.045. In the environmental quality, the sunshine index has the largest weight of 0.032. Equation 6 is the calculation of environmental assessment scores.


(6) $$RI=\overset{k}{\underset{i=k}{\sum }}s_{i}w_{i}\times 100,\left(k=1,2,\cdots ,k\right)$$

In Equation 6, RI represents the environmental assessment score. k represents the number of indicators. $s_{i}$ represents the normalized score of each intermediate layer. $w_{i}$ represents weight.

## 4 Empirical research and optimization strategies for community planning guided by environmental hygiene and public health

As smart cities are gradually built, community environmental health issues become increasingly prominent. To validate the public health oriented community environmental evaluation system proposed in this study, an empirical analysis was conducted using a community in Lanzhou City, Gansu Province, China. Based on the evaluation results, relevant suggestions were proposed for the environmental optimization strategy of the community. According to the experimental results, the research believes that the experimental subjects need to improve the coordination of land use and reading, and the efficient use of land should also be strengthened to realize the centralized layout of various functions.

### 4.1 Empirical analysis results

**Figure 3:**
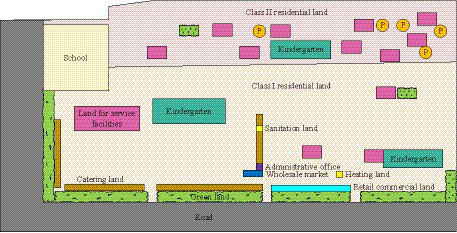
Land use status of the community.

The research object was located near a petrochemical company, and there were three residential communities within the community, with a total population of 8,279 people. The proportion of teenagers, middle-aged people, and older adult people was about 1:1.9:2.5. Among the 8,279 people in the community, 1,546 were adolescents aged 0–14, 2,872 were adults aged 15–64 and 3,861 were older adults aged 65 years and older. There are different needs for the environmental construction of residential communities of different occupations and age groups. For example, the older adult and children want to be equipped with larger and richer outdoor activity space, young people are actively exploring healthy ways to travel, and increasing the parking space of non-motor vehicles is in line with the environmental improvement needs of public health oriented communities. The total area of the community was 0.1151 square kilometers, with abundant commercial, educational, energy facilities, and a large medical institution nearby, which could effectively meet the daily living needs of residents. However, due to the large amount of industrial land around the community, residents were long-term affected by pollutants such as noise, exhaust gas, and wastewater. To gain a detailed understanding of the real situation of the research community, the study conducted a survey in the form of a questionnaire. The questionnaire covers pollution exposure, culture and education, medical and health care, community services, leisure activities, municipal foundation, shopping environment, food environment, sidewalk risk, environmental comfort and aesthetics. In the research community, a total of 273 questionnaires were distributed, 264 questionnaires were collected, 8 invalid questionnaires were excluded, and 256 valid questionnaires remained, with a male to female ratio of 1:1. Figure 3 showed the current land use status of the community.

**Figure 4:**
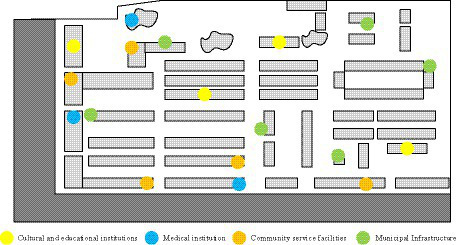
Distribution of public service facilities in the community.

In Figure 3, the land use of the community included residential, public management and public service facilities, commercial service industry facilities, road and transportation infrastructure, public facilities, green spaces, and squares. Among them, there are only 13 land areas for service facilities, except for residential land, accounting for less than 30%. According to the current land use of the research community, the mixed entropy of land use function was calculated to be 0.66, indicating that the current community had a single land type and uneven area distribution. According to the calculation results of the mixed entropy of the land use function, the area proportion of the community is not balanced, and the residential land accounts for 72.50%. The land for public management and public service, commercial service facilities, road and transportation facilities, green space and square land is small, and no industrial land and logistics storage. The proportion of residential area was too large, while other facilities occupied less land, which did not meet the current requirements for the construction of smart cities. The pollution exposure level of the entire community was measured based on the subjective evaluation of community residents. Although there were many original polluting enterprises in the community that had been rectified, residents who had lived for a long time indicated that their health status was still continuously affected. In this study, more than 250 residents collected scores for community pollution exposure, with a score of 1–10; living in high pollution exposure space for a long time with strong inhibition on human health, the lowest score is 1 point. The lower the pollution degree, the higher the score, the pollution degree of the living environment is lower, and their own health is not affected by the pollution exposure, and the highest score is 10 points. The current community pollution exposure was 5.97 points, as calculated by a weighted average. This indicated that although the current research community was less polluted, there was still a risk of exposure. The current population density, building density, and plot ratio of the research community were 719.3 people/hectare, 19.9%, and 1.7, respectively. This indicated that the current population and building density of the community were within the standard range, while the plot ratio was higher than the standard range. Figure 4 showed the distribution of public service facilities in the research community.

**Figure 5:**
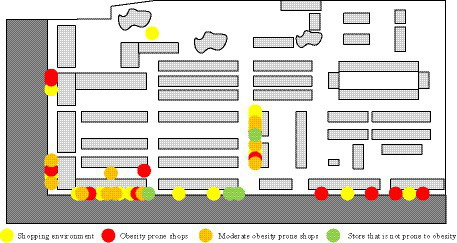
Distribution of the business environment within the community.

In Figure 4, there were three kindergartens and one experimental school in the community, with a service range of 300 meters and 1 kilometer respectively, covering the entire community. However, there was a lack of various activity centers in the community, so the score for cultural and educational facilities was only 3 points. As for medical institutions, there were one health service center, one chain pharmacy, and one traditional Chinese medicine clinic in the community, which could meet the regular medical needs of residents. Therefore, residents gave a score of 8.5 for medical institutions. However, the community service facilities in the research community were relatively backward, so the score was only 2.5 points. In terms of leisure and entertainment, the community had a relatively rich range of leisure activity venues and green parks, so the score for this aspect was relatively high, at 7.5 points. The municipal infrastructure of the community is relatively perfect and in good use, with the large number of garbage cans in the community, emphasizing garbage classification and convenient use; at the same time, the water supply and drainage facilities of the community are relatively perfect, without insufficient water supply and sewage discharge and treatment in the community; so the municipal infrastructure is 8.5 points. Figure 5 showed the distribution of business environment within the research community.

**Figure 6:**
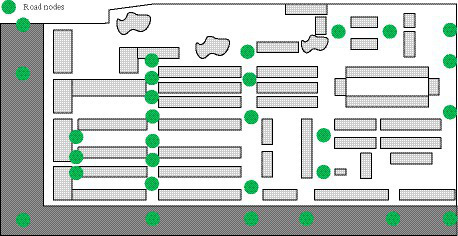
Road nodes of the community.

In Figure 5, both the shopping and food environments in the community were relatively rich, with various shops and restaurants, and a lack of large shopping malls. In addition, due to the influence of residents’ dietary preferences, the consumption frequency of obesity prone foods was relatively high. Therefore, the scores for the shopping environment and food environment were 7 and 6.5, respectively. Figure 6 showed the road nodes of this community.

**Figure 7:**
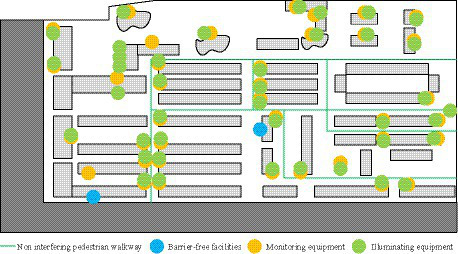
Distribution of community safety facilities.

In Figure 6, there were a total of 31 road nodes, 27 loop roads, and 3 intersections within this community. Therefore, the ring connectivity index and connectivity index of the community road network were 0.39 and 1.67, respectively. Based on the above data, the community’s connectivity and intersection density scores were 7.5 and 5.5, respectively. Due to the close relationship between the connectivity of the road network and the location accessibility, the location accessibility was scored based on the road network structure, residents’ living needs, and the number of service facilities. The accessibility scores for living service facilities and transportation facilities were 6.5 and 8, respectively. Figure 7 showed the distribution of community safety facilities.

In Figure 7, there was a traffic mode in the community that achieved the separation of people and vehicles, with non-interfering sidewalks. However, the occupation of sidewalks was relatively severe, so the road risk score was only 5 points. In addition, there were few barrier free facilities in the community, only tactile paving and barrier free toilets were set, so only 3 points were given for barrier free facilities. There were many monitoring facilities in the community, with a density of 3.83 locations per hectare. But there were fewer installations in public activity areas, so the score for monitoring facilities was not high, at 5.5 points. However, the lighting equipment in the community was widely distributed and in good use, resulting in a high score of 8 points. In terms of comfort, the community’s cleanliness, green configuration, solar exposure index, and resource facility scores were 6, 5.5, 3, and 5, respectively. In terms of aesthetics, the scores for landscape design, disorderly neighborhood environment, and architectural design were 4, 5.5, and 8, respectively. Based on the above results, Table 6 showed the environmental assessment results of the research community.

**Table 6:** Results of the environmental evaluation of the community.

Target layer	Weighted score	The standard layer	Weighted score	Index layer	Weighted score
Land use	3.44	Variety	2.46	Land-use function mix degree	1.69
				Pollution exposure	0.76
		Density	0.99	Density of population	0.33
				Plot ratio	0
				Building density	0.66
Service facility	1.46	Public service	0.95	Education	0.11
				Medical treatment	0.21
				Community service	0.04
				Arder	0.36
				Municipal foundation	0.24
		Business environment	0.51	Shopping environment	0.18
				Food environment	0.33
Place convenience	0.94	Connectivity	0.46	Road network connectivity and loop communication index	0.34
				Intersection density	0.12
		Accessibility	0.47	Service facility	0.29
				Means of transportation	0.18
Environmental quality	0.51	Safety	0.22	Road risk level	0.1
				Barrier-free structures	0.02
				Monitoring equipment	0.06
				Lighting equipment	0.04
		Amenity	0.2	Neatness	0.08
				Green configuration	0.03
				Sunlight index	0.1
				Resources and facilities	0.07
		Beauty	0.1	Landscape design	0.02
				Degree of neighborhood environment disorder	0.05
				Architectural design	0.02

In Table 6, the scores for land use, service facilities, site convenience, and environmental quality were 3.44, 1.46, 0.94, and 0.51, respectively, with a total score of 6.35 for the overall environment. In the aspect of site convenience, the network loop α index is low, and the connectivity β index is higher than 1.5, indicating that the road system has good network formation and convenient passage. However, the proportion of intersections is low, and the residents of non-motor vehicles are highly safe, and the route collocation is inconvenient. At the same time, the accessibility of the food environment is low in the 15 min life circle. In terms of the overall community environment, the score was only 6.35 points. It can be seen that the overall satisfaction of community residents with the environment is not high, and the community environment needs to be improved in terms of land use, service facilities, environmental quality and site convenience. Affected by the above problems, the residents of the community are troubled by insufficient activity and high pollution exposure, which affects the physical health of the residents.

### 4.2 Community planning optimization strategies

**Figure 8:**
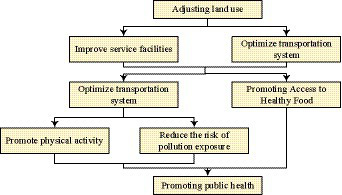
Promotion process of public health.

Due to the low mixing degree of land use, limited public activity space, and ineffective pollution exposure barriers in the community, the physical and mental health of community residents is seriously threatened. Therefore, to improve the health status of community residents, research has proposed a community environment optimization strategy based on public health. Figure 8 shows the process of promoting public health.

According to Figure 8, land use can improve service facilities and transportation system, improve environmental quality, promote physical activity and reduce pollution retention risk, reduce food access and promote public health. This is because as a fundamental problem of the community-building environment, the type and proportion of land use, the equipment of various service facilities, the convenience of internal and surrounding road network or the improvement of internal environment quality, are inseparable from the coordination of land use. At the same time, the efficient use of land allows various functions to be centrally arranged and enriches the diverse usage methods of residents in limited space, creating a convenient, attractive, and high-quality environment that gives residents a sense of belonging. In this case, ensure that the living environment is not negatively affected by the outside world and avoid being exposed to pollution. Residents are more willing to go out and engage in a sports activity, or choose a healthy diet with a positive and happy mood, ultimately promoting their public health. Among the environmental factors that affect public health, the weight of land use accounts for over 50%, indicating its most profound impact on public health. By balancing the proportion of various types of land and planning community spaces reasonably, public health levels can be effectively improved. Therefore, in response to the land use issues in the research community, it is proposed to connect the isolation walls of the three residential areas within the community to form an open and diverse community. And land for public management and public service facilities are increased, as well as land for public facilities. The location of the community service center is moved, placed in the community center, and various activity centers are integrated on the same building to improve land use efficiency. The commercial environment is regulated in this experiment to form a more dynamic commercial space. Specific measures are as follows: increase the parking lot in the community 0.22 hectares, administrative office land 0.01 hectares, adjust the land location, the community service center in the community, surrounding and kindergarten, small green space, catering shops form activity center, the older adult activity room, comprehensive cultural activity center, reading room and other functions distributed on the same building, improve the efficiency of land use. The catering and retail stores around the vegetable market are well-organized to form a continuous space full of commercial competitiveness. Table 7 shows the land use indicators before and after optimization.

**Table 7:** Land use indicators before and after optimization.

Type	Land use ratio/%		Type	Land use ratio/%		Type	Land use ratio/%	
	Before optimization	After optimization		Before optimization	After optimization		Before optimization	After optimization
Residential land	72.5	72.3	Class I residential land	52.3	52.8	Residential land	48.1	48.7
						Land for service facilities	4.2	5.9
			Class II residential land	20.2	19.5	Residential land	16.2	15.8
						Land for service facilities	4.0	5.4
Land for public administration and service use	3.3	3.4	Administrative office land	0.05	0.2			
			Land for education and scientific research	3.2	3.2	Primary and secondary school land	3.2	3.2
			Medical land	0.04	0.04	Hospital land	0.04	0.04
Land use for the commercial services industry	1.3	1.5	Trade estate	1.3	1.5	Retail land	0.3	0.8
						Food and beverage land	0.9	0.7
Land used for roads and transportation facilities	20.2	20.2	Land for roads	20.2	20.2			
Land for public facilities	0.1	0.2	Land for supply facilities	0.06	0.1	Land for heating facilities	0.06	0.1
			Land for environmental facilities	0.04	0.1	Sanitation land	0.04	0.1
Green space and square land	2.7	2.5	Green area for environmental protection	2.7	2.5			

According to Table 7, the proportion of residential land decreased from 72.5 to 73.2%, the proportion of public management and service land increased from 3.3 to 3.4%, the proportion of commercial service land increased from 1.3 to 1.5%, the proportion of public facilities land increased from 0.1 to 0.2%, and the proportion of green land and square land decreased from 2.7 to 2.5%. In terms of the convenience of the site, improving the connectivity and density of the road network can encourage residents to choose non-motorized vehicles, increase physical activities, and reduce the physical quality of the residents, improve the accessibility of the site, reduce the difficulty of obtaining healthy food, and finally realize the purpose of promoting public health. In addition, by increasing the density of intersections, it can also increase the connectivity between community roads and urban main roads, and enhance the diversity of travel modes. In terms of environmental quality, to ensure the community safety, the proportion of undisturbed sidewalks is increased, and reasonable traffic control is implemented. And the ratio of monitoring and lighting equipment is increased to ensure public safety and reduce the occurrence of criminal activities. The specific plan is: add 8 monitoring equipment, so that the number of monitoring equipment reaches 52 places, and the density is 4.52 places / ha. Lighting equipment was increased to 60 places, with a density of 5.21 places / ha. In addition, to reduce the road risk, various facilities installed on sidewalks will be removed to improve the road smoothness. Accessible facilities are added to provide a good living environment for people with disabilities. To improve the community comfort, greenery is added within the community to provide comfortable leisure space for residents while reducing pollution exposure. By increasing the greening facilities, the purification characteristics of the plants themselves can be utilized to reduce the pollutants in the community, thus reducing the pollution exposure of the residents and promoting public health. Considering the lack of various activity centers in this community, which makes it difficult to meet the daily activity needs of residents, increase activity places in the community to promote physical activity and improve the physical quality of residents.

## 5 Conclusion

As urbanization advances, the current public health crisis faced by society becomes increasingly severe. To explore the relationship between community environment and public health, the study constructs an environmental health assessment system using 5Ds theory and AHP. To validate the evaluation system, an empirical analysis is conducted using a community in Lanzhou City as an example. These experiments confirms that the scores for land use, service facilities, venue convenience, and environmental quality in the community are 3.44, 1.46, 0.94, and 0.51, respectively, with a total score of 6.35 for the overall environment. The scores for land use, service facilities, site convenience and environmental quality were 3.44, 1.46, 0.94, and 0.51, respectively, and the overall environmental score was only 6.35. The community has problems such as poor diversity of land use types, high risk of pollution exposure, difficulty in obtaining healthy food, and low green allocation, which seriously affect the health of local residents. To solve the above issues, research proposes optimization suggestions from improving the richness of land use types, optimizing transportation systems, improving environmental quality, and improving service facilities. By reducing the proportion of residential land use, increase the proportion of other land types such as the use of land for public service facilities and commercial land, and improve the diversity of community land use. Optimize the community transportation network to reduce food access and increase green plants to reduce pollution exposure. After improvement, the proportion of residential land decreased from 72.5 to 73.2%, the proportion of public management and service land increased from 3.3 to 3.4%, the proportion of commercial service land increased from 1.3 to 1.5%, the proportion of public facility land increased from 0.1 to 0.2%, and the proportion of green land and square land decreased from 2.7 to 2.5%; the diversity of land use improved significantly. It effectively promotes the physical activity of the residents, reduces the pollution exposure and the difficulty of obtaining healthy food, and effectively promotes the healthy life of the community residents. Although this study conducted a comprehensive exploration of the relationship between the community environment and public health, it only considered life and did not consider the effects of work and commuting on residents’ health, thus having some limitations. At the same time, only the typical unit community was selected as the research object, and the relevant research on the commercial housing community and the urban village community has not been carried out, so the universality of the research is relatively lacking. In the future, research on different types of communities can be carried out to further explore the positive response plan of rapid urbanization on community public health, and fully consider the influencing factors such as commuting, so as to make the optimization strategy more practical.

## Data availability statement

The original contributions presented in the study are included in the article/supplementary material, further inquiries can be directed to the corresponding author.

## Author contributions

FC: Conceptualization, Supervision, Writing – review & editing. WS: Investigation, Writing – original draft. FZ: Investigation, Writing – original draft. MG: Investigation, Methodology, Writing – original draft. CW: Formal analysis, Investigation, Writing – original draft. GL: Investigation, Writing – original draft. LW: Investigation, Writing – original draft. YW: Writing – original draft. CZ: Investigation, Writing – original draft.
